# Local structure and paramagnetic properties of the nanostructured carbonaceous material shungite

**DOI:** 10.1186/s11671-015-0767-9

**Published:** 2015-02-19

**Authors:** Serhii Volodymyrovich Krasnovyd, Andriy Andriyovich Konchits, Bela Dmytrivna Shanina, Mykhaylo Yakovych Valakh, Igor Bogdanovich Yanchuk, Volodymyr Olexsandrovych Yukhymchuk, Andriy Volodymyrovich Yefanov, Mykola Andriyovich Skoryk

**Affiliations:** V.E. Lashkaryov Institute of Semiconductor Physics NAS of Ukraine, 03028 Kyiv, Ukraine; Nanomedtech LLC, 68 Gorkogo str, 03680 Kyiv, Ukraine

**Keywords:** Shungite, Nanostructured carbonaceous material, Electronic properties, Oxygen-deficient *E*'_γ_ centers, 81.05.U-, 76.30.-v, 73.61.Ph

## Abstract

Using a scanning electron microscopy, elemental analysis, electron paramagnetic resonance, and Raman scattering methods, two types of the shungite materials (Sh-II from Zazhogino deposit and shungite from a commercial filter (ShF)), with different carbon content and porosity, are studied in this work. It was established by scanning electron microscopy data that the structure of the shungite samples is formed by a micron-size agglomeration of carbon and silicon dioxide clusters. It is found from the Raman data that carbon fraction is formed from sp^2^-hybridized clusters, size of which increases from 9 up to 12 nm after annealing of the samples. High conductivity of shungite is found to belong to the carbon nanoclusters of different sizes. Big clusters give the conduction electron spin resonance signal with a Dysonian line shape with variable *g*-factor and line width.

The careful search of the nature of two other narrow electron paramagnetic resonance signals in shungite, which used to be prescribed to fullerene-like molecules, is fulfilled. Here, it is shown that the oxygen-deficient *E*'_γ_ centers are responsible for these signals. A strong correlation is revealed between the concentration of *Е*'_γ_ centers and the line width of conduction electron spin resonance signal, which occurs under annealing process of the samples at *T* = 570 K. The correlation reasons are a spin-spin coupling between two spin subsystems and time dependent of the *Е*'_γ_ concentration during annealing process.

## Background

Shungites are carbon-rich rocks of Precambrian age widespread over Karelia (Russia). This is heterogeneous materials consisting mainly of amorphous silicon dioxide and carbon, with nanocarbon content from 5% up to 98%. The shungite organic matter is represented by a non-crystalline and non-graphitized form of carbon [1].

Due to its abundance and unusual properties, shungite has the great potential for application in technology and human life [1]. In particular, shungite shows unique electrochemical properties due to high resistance to acids. The most intriguing is the question of the presence of fullerenes in natural shungite [1-5]. The discussion on this question continues, but some researchers propose to use a shungite as a raw material for fullerene preparation [6,7].

Unlike a raw coal, shungite is characterized by a stability of its properties. It was marked that this material is not inclined to graphitization [1,8]. Nevertheless, the real local structure of carbon in shungite is insufficiently studied. Common opinion concerning shungite nanocarbon (SHNC) structure worked out up to now from X-ray and electron diffraction study [9,10] can be summarized as follows. SHNC presents a multilayer globular structure with the average size of globs about 10 nm. Graphite-like layers are skewed so that the hexagonal symmetry is lowered to trigonal. It is a basis for the supposition about fullerene-like structure of the globs [10]. Significant changes in the porosity of shungite samples were observed after transformation into aqueous dispersion and subsequent drying [11].

Due to its specific physical and chemical properties, shungite is widely used in metallurgy, water purification, thermolysis, etc. Shungite is also a sorbent for removal of many pathogenic bacteria and heavy metals from contaminated water.

Useful practical properties of the shungite are known for a long time, but there are no any scientific reasons for answering the questions, why this mineral produces a medicinal effect and what special features emerge in the shungite in contact with an organic material. First of all, we need to understand the connection between the microstructure of shungite and its physical properties, namely, electron structure, conductivity, magnetic properties, and appearance of different defects with temperature and pressure change.

In this work, the shungite samples with different nanocarbon contents [Sh-II (Zazhogino deposit) and shungite from commercial filter (ShF)] were studied by scanning electron microscopy (SEM), energy dispersive X-ray (EDX) elemental analysis, electron paramagnetic resonance (EPR), and Raman scattering (RS) methods.

The main goal of our research is to understand the correlation between the shungite morphology, features of local structure, and electronic properties including formation of different defects with temperature and pressure treatment.

## Methods

### Sample preparation

Samples of the Sh-II and ShF varieties with nanocarbon content 25 to 80 at.% were cut to the sizes of approximately 3 × 2 × 2 mm^3^. In our experiment, the samples of both types were subjected to a heat treatment and pumping out.

### Characterization

Electron microscopy of heterogeneous shungite samples was carried out using the high-resolution SEM TESCAN MIRA 3 MLU (TESCAN ORSAY HOLDING, Brno-Kohoutovice, Czech Republic); the elemental composition was determined with energy dispersive X-ray spectrometer X-max (Oxford Instruments, Abingdon, England). The EPR spectrometer “Radiopan” SE/X-2244 (Radiopan Firm, Poznan, Poland) with 100-kHz modulation of the magnetic field was used to measure *g*-factor values, line width *ΔH*_*pp*_, and spin concentration *N*_*s*_. Raman spectra were produced by Ar-Kr laser with a wavelength, *λ* = 405 nm, and registered at room temperature by means of spectral complex Jobin Yvon T64000 (Horiba Jobin Yvon International SAS., Longjumeau, France).

## Results and discussion

### Scanning electron microscopy

**Figure 1:**
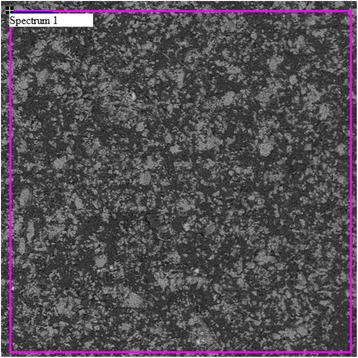
**The surface of the shungite sample Sh-II-1.**

Figure 1 shows the surface of a typical shungite sample Sh-II-1. It is seen in Figure 1 that the surface of the sample Sh-II-1 consists of micron-size aggregates of clusters.

The elemental content of the sample Sh-II-1 obtained from SEM/EDX data is presented in Table 1. Analysis shows that the shungite structure is formed by clusters of carbon and silicon dioxide, including small particles of pyrite (FeS_2_), iron oxide, and aluminum oxide.

**Table 1:** **Element composition on a surface of the shungite Sh-II-1 sample (at.%)**

**Elements**	**C**	**O**	**Si**	**Fe**	**S**	**Al**
Spectrum 1	79.8	14.83	4.45	0.04	0.05	0.51

It is seen in Figure 1 that the specific area (spectrum 1) has a high concentration of carbon, approximately 79.8%, and small amount of silicon and aluminum. The high content of oxygen (approximately 14.83%) in this area indicates the existence of oxygen atoms not only in silicon dioxide and aluminum structures but also in carbon clusters. In Figure 1, clusters of silicon dioxide are gray colored and carbon clusters are marked with the black one. Also, there are small inclusions of light color corresponding to iron clusters. They may be of both types of particles such as pyrite or iron oxide. Therefore, the studied shungite samples were consist of a carbon and silicon dioxide mixture with inclusions of iron compounds.

The sample ShF1 also was investigated by SEM/EDX and showed results similar to that of the sample Sh-II-1 but with a high content of iron and less homogeneous mixing of carbon and silica phases.

### Raman scattering

**Figure 2:**
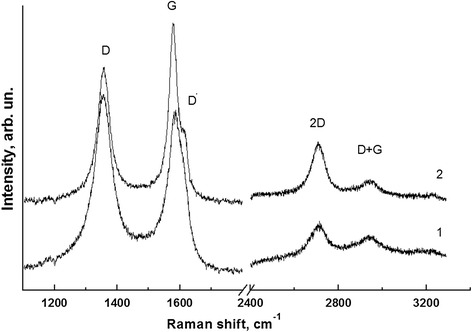
**Raman spectra of the Sh-II-1 sample.** 1 - before annealing, 2 - after annealing at temperature 550^о^С during 0.5 h.

The Raman spectra of the sample Sh-II-1 across the range from 1,000 to 3,300 cm^−1^ were investigated. Figure 2 presents the first and second order spectra obtained from the initial sample Sh-II-1 (curve 1) and after annealing at *T* = 550°C (curve 2). Measurements were done in one of the carbon-rich area of this sample (in silicon-rich areas, RS signals have a lower intensity).

It is known that G band (approximately 1,583 сm^−1^) is related to the twofold-degenerated mode of *E*_2g_ symmetry in the center of the Brillouen zone, and it is a manifestation of the extended oscillations of all atoms with sp^2^ bonds in the benzol rings. D band (approximately 1,350 сm^−1^) [12] is a breathing mode of *A*_1g_ symmetry that involves LO phonons near the K-point of Brillouin zone [13,14]. They also observed a high-frequency shoulder at a G band - so-called D' band.

The general view of spectra and parameters (Table 2) confirms that the shungite compound is formed with well-ordered sp^2^-hybridized carbon nanoclusters.

**Table 2:** **Spectral characteristics of the Sh-II-1 sample before and after annealing**

***n*** **/** ***n***	***ν***_***D***_ **(cm** ^**−1**^ **)**	***Г***_***D***_ **(cm** ^**−1**^ **)**	***ν***_***G***_ **(cm** ^**−1**^ **)**	***Г***_***G***_ **(cm** ^**−1**^ **)**	***I***_***D***_ **/** ***I***_***G***_	***L***_***a***_ **(nm)**	***ν***_***D*′**_ **(cm** ^**−1**^ **)**
Initial	1,355.8	75.7	1,583.4	59.2	1.47	9.3	1,612.8
Annealed	1,357.0	49.0	1,578.3	34.1	1.09	12.5	1,614.8

After annealing, narrowing of D and G bands and redistribution of their intensities were observed (Figure 2). This behavior is fairly typical for sp^2^-hybridized carbon structures when annealing promotes structure ordering. Using the empirical formula [15] for estimation, the size of cluster is:

1 $$ {L}_a\left(\mathrm{nm}\right)=560/{E_{\mathrm{l}}}^4{\left({I}_d/{I}_G\right)}^{-1}, $$

where *E*_l_ - Raman spectrum excitation energy, and we found that during the annealing, the size of nanoclusters increased from 9.3 to 12.5 nm. This is a typical process of consolidation of carbon clusters where the number of defects decreases and the structure becomes more perfect.

In addition, one can also conclude that the presence of silicon dioxide clusters does not affect significantly the processes of temperature transformation of shungite carbon structure.

### Electron paramagnetic resonance

**Figure 3:**
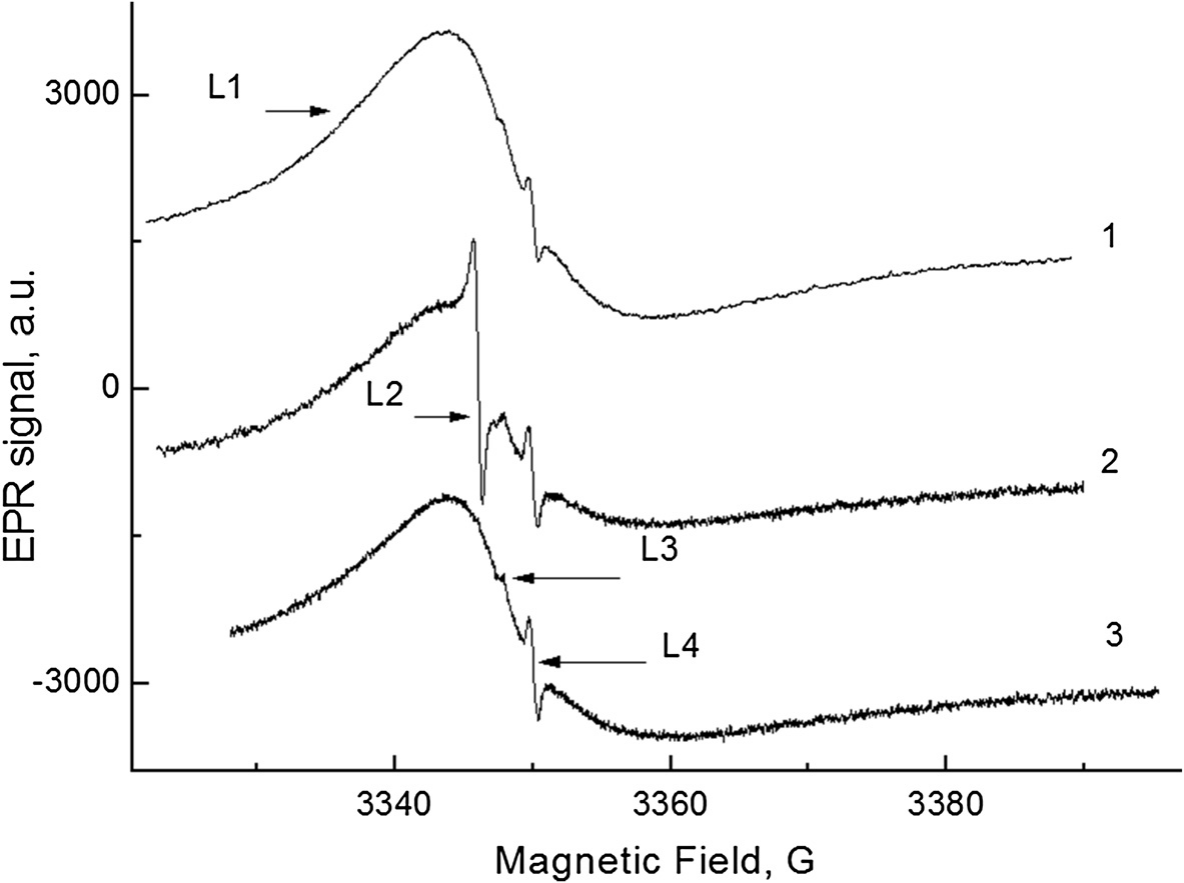
**The EPR spectra of the ShF1 sample at different conditions.** 1 - initial, 2–0.5 h pumping at 130°C, and 3 - after 24-h storage in air. *ν* = 9,380 MHz. *T*meas. = 300 K.

EPR spectrum of the shungite samples consists of four resonance signals with different line widths, *g*-factors, and integral intensities. The EPR spectra of sample ShF1, recorded under different conditions, are shown in Figure 3.

It should be noted that lines L1, L3, and L4 with varying intensity are observed in most of the samples. The line L2 emerges only in some samples (in about 20% ones), mainly after vacuum annealing at *T* = 120°C ÷ 300°C.

Let us consider in detail the origin and characteristics of these EPR lines. The most intensive line L1 has asymmetrical shape (so-called Dyson line shape) in all the samples including ShF1 (Figure 3), which demonstrates relation to electron conductivity and indicates a high conductivity of the samples due to nanocarbon [16]. This is an important fact that follows directly from the data of Figure 3.

**Figure 4:**
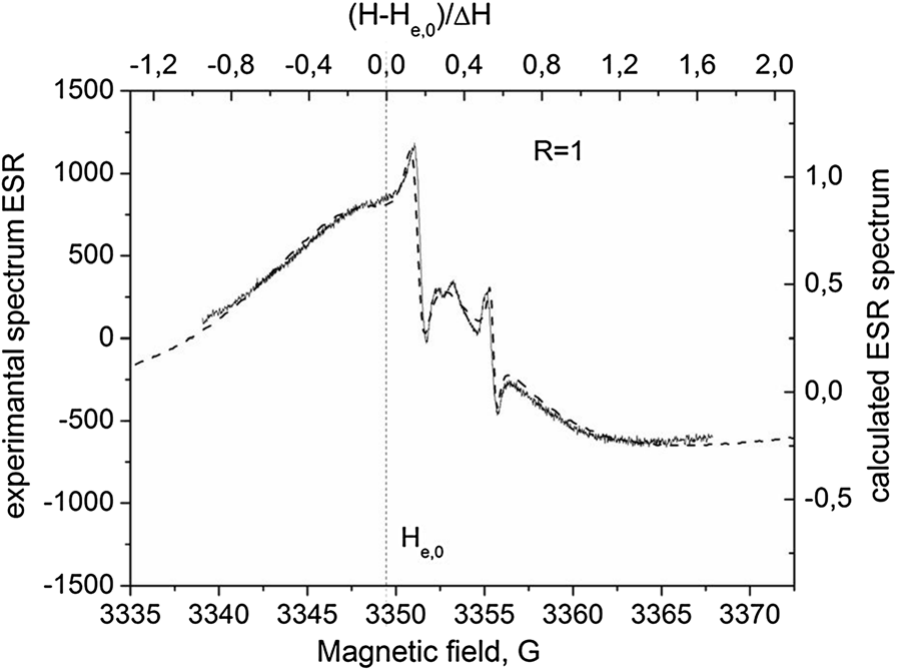
**Theoretical description of the experimental spectrum (Figure** **3** **, curve 2).** The dotted line indicates the position *H*_res_ for conduction electrons.

In describing the experimental spectrum, as it is shown in Figure 4, we used the theoretical expression, given in [17,18] for condition *d* ≫ *δ*, *δe*, where *d* is the thickness of the sample, *δ* is the skin layer thickness determined by the conductivity and the microwave field frequency of the sample, and *δe* is the electron diffusion path for spin relaxation time *T*_2_. In this case, the EPR line shape is determined only by a single parameter *R*^2^ = *T*_*D*_/*T*_2_ = (*δ*/*δe*)^2^. The resonance field *H*_res_ does not coincides with position at the magnetic field *H*, where amplitude of the derivative of absorption signal is equal to zero, as soon as a signal is a combination of absorption and dispersion. Consequently, *g*-factor of free electron can be found only after fitting the calculated spectrum to the experimental one. By this reason, *H*_res_ is determined only after the fitting as a point at the *H* axis, which corresponds to point zero at the upper dimensionless axis in Figure 4.

As a result, we find:

$$ g=2.0030\pm 1\times 1{0}^{-4};\ R=1;\varDelta {H}_1=16.5\ \mathrm{G}. $$

The nature of the symmetrical line L2 is probably associated with small carbon clusters (*d* ≪ *δ*, *δe*) for which the spin resonance line has the Lorentzian shape, but not the Dysonian one. Its parameters are *g*_2_ = 2.0029; *ΔH*_2_ = 0.76 G. The sensitivity of the line L2 to pumping out the oxygen (like coal [19]) shows that small carbon clusters are contact with molecular oxygen due to the presence of open nanopores in the sample ShF1.

Lines L3 and L4 show a correlated behavior as lines of separate spin system. The intensity of lines L3 and L4 increases during vacuum annealing at *T* > 120°C (Figure 3, curve 2). Using the reference sample MgO: Cr^3+^, we determined the spin concentration of the centers which form the L3 and L4 spectral lines: *Ns* ≅ 0.8 × 10^16^ cm^−3^. Parameters of these lines (*g*_3_ = 2.0018, *ΔH*_3_ = 1.0 G; *g*_4_ = 2.00055, *ΔH*_4_ = 0.4 G) and their behavior during the annealing are very similar to the behavior of the EPR lines observed at oxygen deficiency in silicon dioxide (so-called *Е*'_γ_ centers) [20]. So the lines L3 and L4 can be *Е*'_γ_ center spectrum components with anisotropic *g*-factor in non-crystalline matter and the absorption peaks in the *g*_II_ and *g*_⊥_ orientation.

**Figure 5:**
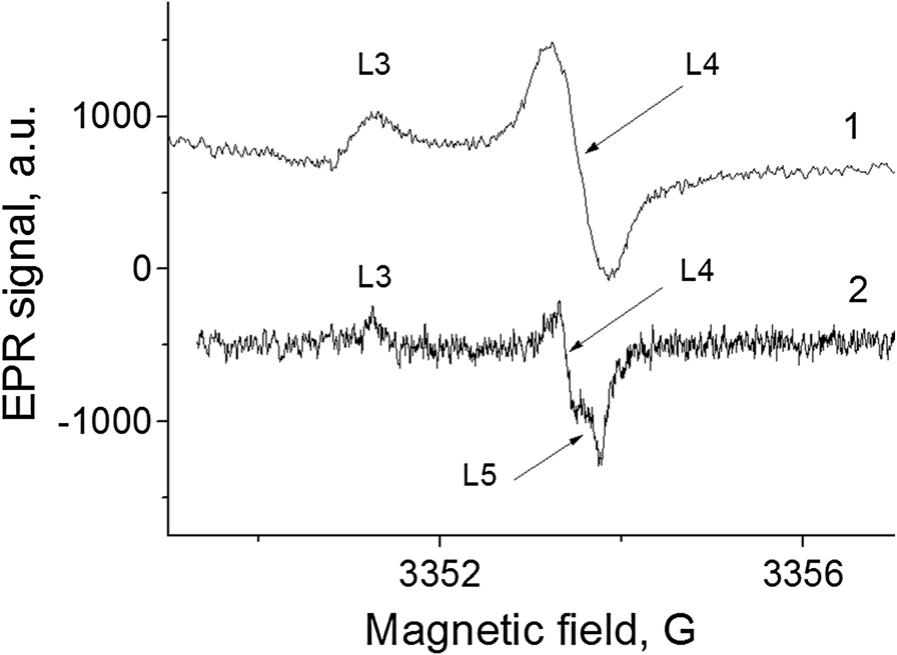
**EPR spectra of** ***Е***'_**γ**_ **centers in the pumped off sample Sh-II-1.** (1) - *P*_mw_ = −32 dB, *U*_mod_ = 0.05 G. (2) - *P*_mw_ = −37 dB, *U*_mod_ = 0.01 G (accumulation, *n* = 9). *ν* = 9,390 MHz.

In fact, line L4 consists of two narrow lines L4 and L5 with effective *g*-factors of 2.0006 and 2.0003, as it is seen in Figure 5 at a significant decrease of the modulation amplitude. Parameters of the L3 and L4 lines precisely coincide with parameters of *Е*'_γ_ centers with the anisotropic *g*-tensor (*g*_1_ = 2.0018, *g*_2_ = 2.0006, and *g*_3_ = 2.0003), which are induced in silica by γ-irradiation [20,21]. It means, that the lines L3 and L4 (Figures 3 and 5) belong to the oxygen-deficiency centers formed in silicon dioxide fraction of shungite. Note that, up to now, the presence of such lines in shungite was associated with the expected presence of fullerene molecules in shungite samples [22].

**Figure 6:**
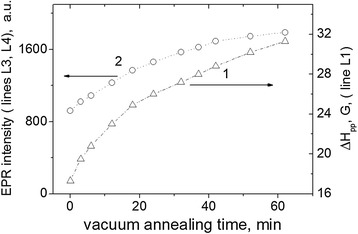
**Vacuum annealing effect Sh-II-1 sample.** The line width of signals L1 (1) and intensity of signals L3, L4 (2), *T*ann = 310°C.

We found a certain correlation between the characteristic parameter lines L1 on the one hand and L3 and L4 on the other hand. Curves in Figure 6 demonstrate the correlated behavior between amplitudes L3 and L4 and line width L1 during vacuum annealing of the sample Sh-II-1 at *T* = 310°C. During vacuum annealing, the intensity of the lines L3 and L4 increases gradually, and at the same time, Dyson line L1 is broadened with a corresponding decrease in its amplitude. Integral intensity of line L1 stays constant during the annealing.

**Figure 7:**
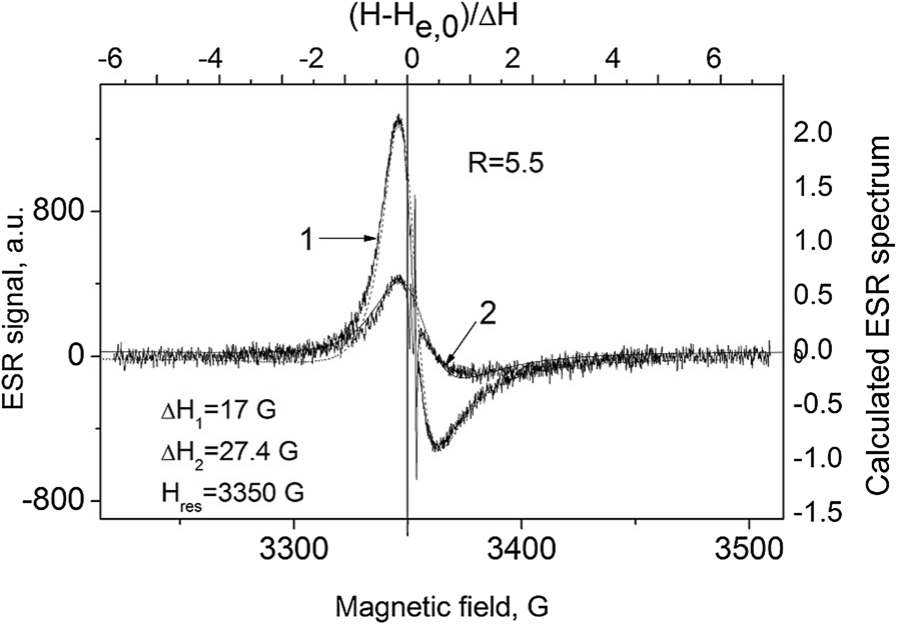
**The ESR spectra of the Sh-II-1 sample.** 1 – initial, 2 - after annealing at T_ann_ = 310°C during 1 h**.** Dotted lines are theoretical description of Dyson lines origin from conducting electrons. *ν* = 9,389.75 MHz.

Figure 7 shows the ESR spectra of the sample Sh-II-1 before and after annealing and the theoretical description according to [17,18].

It can be seen that annealing changes the ESR line width; however, the *R* value remains the same (*R* = 5.5) and different from that of the sample ShF1 (see Figure 4).

## Discussion

Dyson shape of the conduction electron spin resonance (CESR) points out that the shungite samples contain a high concentration of conduction electrons which belong to the conductive areas. It means that some conductive carbon areas are the clusters with ratio *δ*/*d* ≪ 1, where *d* is the size of the cluster. It is known that the most conductive material among the carbons is graphite [23]. The conductivity of the pure graphite flakes is about 3 × 10^2^ (Ohm × cm)^−1^ along axis *c* and 100 times more in basal plane. By this conductivity, the depth of the skin layer is anisotropic with a minimum value *δ*_min_ ≈ 10^−6^ cm along the basal plane and *δ*_max_ ≈ 10^−4^ cm in direction ⊥ to *c* [23]. These facts point to the size of carbon clusters, responsible for CESR, *d* ≫ 10^−6^ cm, i.e., d ≫ 10 nm. The nanocluster sizes of shungite estimated from Raman data are *d* ~ 10 nm. We believe that signal L2 in Figure 3 is a signal from clusters with *d* < 10 nm. In the smaller clusters about 1 ÷ 3-nm electrons undergoes confinement, the energy gap increases and the cluster losts graphite properties. Electrons located at the carbon dangle bonds in small clusters contribute microwave absorption also to the signal L2.

It is revealed that, in this study, a correlation between the line width of signal L1 (conduction electrons in carbon areas) and the integral intensity of signals L3 and L4 (*Е*'_γ_ centers located at interface) is shown in Figure 6.

The dependence on the annealing duration for both values is described by the following functions:

2 $$ {I}_{E'\upgamma}(t)={I}_{E'}\left(t=0\right)+\delta I\cdot \left(1- \exp \left(-t/\tau \right)\right);\kern0.75em \to \varDelta {H}_e(t)=\varDelta {H}_e\left(t=0\right)+\delta \varDelta {H}_e\cdot \left(1- \exp \left(-t/\tau \right)\right) $$

$$ {I}_{E'\upgamma}\left(t=0\right)=950\ \mathrm{a}.\mathrm{u}.;\delta I=980\ \mathrm{a}.\mathrm{u}.;\kern0.75em \to \varDelta {H}_e\left(t=0\right)=16\ \mathrm{G};\delta \varDelta {H}_e=17.5\ \mathrm{G}; $$

$$ {\tau}^{-1}=5.3\times 1{0}^{-4}{\mathrm{s}}^{-1}, $$

where *τ*^−1^ is the rate with which the *I*_*Е*'γ_(*t*) and *ΔH*_*e*_(*t*) are changed depending on the annealing duration. One can see that the increase of *Е*'_γ_ center concentration and the CESR line width is caused by the same process. The line width of CESR is determined by two factors - by the spin relaxation time *T*_2_ and by parameter *R*^2^ = *T*_*D*_/*T*_2_, where *T*_*D*_ is a time of electron diffusion through the skin layer. With the *R* increase, the line width increases insignificantly (about 30%) [23], so that the line width is determined, mainly, by *Т*_2_^−1^. If the annealing at *T* = 600 K causes an increase of *Е*'_γ_ center concentration, the scattering of the conduction electron spins on the spins of *Е*'_γ_ centers due to spin-spin interaction makes *T*_2_ shorter and depending on the *Е*'_γ_ center concentration. Therefore, time dependence (2) is the property peculiar to *Е*'_γ_ center formation. The rate of *Е*'_γ_ center formation is comparable with the rate of the local strain relaxation. The annealing at *T* = 600 K turns off oxygen atom from SiO_2_ surface in the interface between carbon and SiO_2_ clusters; thereafter, the excited charged defect has to relax before becoming an *Е*'_γ_ center. In this reason, the probability of *Е*'_γ_ center appearance *P*_*Е*'γ_ is proportional to *P*_*Е*'γ_ = *W* × exp (−*t*'/*τ*) *dt*', where *W* is a probability of oxygen atom away. During period (0; *t*), the concentration of *Е*'_γ_ changes as the value:

3 $$ {\displaystyle \int {P}_{E\hbox{'}\upgamma}}\left(t\hbox{'}\right)dt\hbox{'} = W\times {\displaystyle \int {P}_{E'\upgamma}}\left(t\hbox{'}\right)dt\hbox{'} = W\times {\displaystyle {\int}_0^t \exp \left(-t\hbox{'}/\tau \right)\kern0.1em dt\kern0.22em \hbox{'}=}\delta I\times \Big(1- \exp \left(-t/\tau \right)\ \mathrm{with}\ \delta I = W\times \tau $$

which corresponds to the experimental function (2).

## Conclusions

SEM/EDX data show that the structure of the investigated shungite samples is formed by a cluster mixture of carbon and silicon dioxide including pyrite (FeS_2_), iron oxide, and aluminum oxide particles.

Analysis of the Raman spectra results to conclusion that nanocarbon structure component in shungite is formed from sp^2^-hybridized, well-ordered carbon clusters. Annealing of the samples leads to the enlargement of carbon clusters, and their structure becomes more perfect.

Asymmetrical spin resonance signal (Dyson line shape) in the EPR spectrum shows that the line L1 belongs to free electron in the nanocarbon areas of the samples with a high conductivity. We have reported for the first time about narrow symmetric signal L2 from nanoclusters. The characteristics of L1 and L2 are determined based on the theory of spin resonance of conduction electrons.

For the first time, origin of spectral lines L3 and L4 was found to be the oxygen-deficient *E*'_γ_ centers. Earlier this signal was associated with the expected presence of fullerene molecules in shungite samples.

The correlation between the intensity of *E*'_γ_ spectra and the broadening of CESR signal after the annealing of the sample was established. The intensity and line width is explained by the process of the strain relaxation during formation of *E*'_γ_ centers near the SiO_2_/carbon interface which is the reason of the observed correlation in time dependence.

## Abbreviations

SHNC: Shungite nanocarbon
SEM: Scanning electron microscopy
EDX: Energy dispersive X-ray spectroscopy
RS: Raman scattering
EPR: Electron paramagnetic resonance
ESR: Electron spin resonance
CESR: Conduction electron spin resonance
